# Ocular toxicity of intravitreal melphalan for retinoblastoma in Chinese patients

**DOI:** 10.1186/s12886-019-1059-4

**Published:** 2019-02-26

**Authors:** Kang Xue, Hui Ren, Fengxi Meng, Rui Zhang, Jiang Qian

**Affiliations:** Department of Ophthalmology, Eye, Ear, Nose, and Throat Hospital of Fudan University, Shanghai Key Laboratory of Visual Impairmentand Restoration of Fudan University, Shanghai, 200031 China

**Keywords:** Retinoblastoma, Intravitreal melphalan, Ocular toxicity

## Abstract

**Background:**

To evaluate the efficacy, complications, and clinical characteristics, including the ocular toxicity, of intravitreal melphalan(IVM) treatment for vitreous seeding in Chinese retinoblastoma patients.

**Methods:**

This was a retrospective, non-comparative analysis including 30 consecutive eyes of 23 patients with viable persistent or recurrent vitreous seeding following retinoblastoma treatment. All of the eyes received IVM injections (20–33 μg). Vitreous seeding control, determination of the ocular toxicity, and the clinical characteristics of intravitreal melphalan treatments were observed.

**Results:**

The mean patient age at the time of the injection was 28 months (median = 22 months, range = 12–50 months). In total, 80 injections were administered in 30 eyes, the overall enucleation-free survival rate was 83.3% (25/30). The complications included retinal pigment epithelium (RPE) and choroidal atrophy (19/30, 63.3%), pupillary synechiae (13/30, 43.3%), iris atrophy (12/30, 40%), retinal vascular occlusion (12/30, 40.0%), optic atrophy (6/30, 20%), vitreous hemorrhage (3/30, 10%), persistent hypotonia and phthisis bulbi (4/30 13.3%), and cataracts (8/30, 26.6%). Twelve eyes demonstrated grade 3 or greater IVM-associated retinal or anterior segment toxicity post injection. Mean dosage given showed significant difference between the groups. There were no significant differences in the retinal toxicity grades regarding the seed classification or seed regression patterns.

**Conclusions:**

Intravitreal melphalan is an effective treatment for refractory vitreous seeding from retinoblastoma, but exhibits both anterior and posterior segment toxicity in Chinese patients.

## Background

Eye preservation and tumor control in patients with retinoblastoma seeding are challenging, despite the advances in the treatment modalities [1, 2]. The intravitreal injection of melphalan is being used more often in the treatment of vitreous retinoblastoma seeds, saving many eyes that once would have been enucleated [3–5]. Previously, Smith et al. [6] reviewed the ocular complications of intravitreal melphalan, while several authors [7–10] reported the complications of intravitreal injections. In addition, Shields [11] and Munier et al. [12, 13] reported minimal toxicity and complications with a 20 to 30 μg dose of intravitreal chemotherapy using melphalan. However, most previous articles reported results from Caucasian populations in Europe and America [7–10], with few articles from Asian populations. We found higher hates of complications than previously reported. Herein, we reported our experience with intravitreal melphalan in treating vitreous seeding in 30 eyes of 23 Chinese retinoblastoma patients to evaluate the efficacy, complications, and clinical characteristics, including the ocular toxicity.

## Methods

### Ethics approval and consent to participate

The Institutional Review Board at Eye, Ear, Nose, and Throat Hospital of Fudan University approved this study. This was a retrospective, non-comparative analysis. After the risks for extraocular extension, vitreous hemorrhage, retinal detachment, and intraocular infection were explained; written informed consent was obtained from the parents, caretakers, or guardians on behalf of all of the children, and placed in the patient record.

### Patients

The medical records of 23 patients (30 eyes) diagnosed with refractory vitreous seeding from retinoblastoma at the Department of Ophthalmology at the Eye, Ear, Nose, and Throat Hospital of Fudan University in Shanghai, China, from March 2014 to September 2016, were reviewed for this study. The inclusion criteria were eyes with viable vitreous retinoblastoma seeds that were persistent or recurrent following the standard treatment methods. The exclusion criteria were eyes that displayed an additional viable solid intraretinal retinoblastoma or viable subretinal seeds, or those deemed at risk for metastatic disease with uveal or optic nerve invasion [10].

### Treatment

The protocols for systemic chemoreduction and intra-arterial chemotherapy have been published previously [14, 15]. Systemic chemoreduction dose was intravenous vincristine 0.05 mg/kg for patients≤10 kg, 1.5 mg/m2 for patients >10 kg 1 day, etoposide 5 mg/kg for patients ≤10 kg, 150 mg/m2 for patients >10 kg × 2 day and carboplatin 18.6 mg/kg for patients≤10 kg, 560 mg/m2 for patients >10 kg 1 day [14].The intra-arterial chemotherapy consists of melphalan 3, 5 or 7.5 mg, increasing with tumor size and the patient’ s age, while the carboplatin dose was 20 mg [15].

The intravitreal injections were performed under general anesthesia. Before each injection, the locations of the retinal tumor and vitreous seeds were determined using RetCam III(Clarity Medical Systems, CA) and an indirect ophthalmoscope. Massaging the eyeball decreased the intraocular pressure. All of the eyes received intravitreal melphalan injections via the transconjunctival pars plana route, with concomitant triple-freeze cryotherapy at the injection site during the needle withdrawal for the prevention of extraocular seeding. The eyeball was moved gently with the forceps, back and forth, to cause drug dispersion throughout the vitreous cavity, and preferably, to the site of the vitreous seeds [16]. Topical corticosteroid/antibiotic ointment was applied immediately post operatively.

We assessed response to treatment and complications under anesthesia every 2–4 weeks with RetCamIII and indirect ophthalmoscope. UBM was not used routinely. When final seed regression pattern is noted [12], which indicated control of vitreous seeds, the intravitreal injection would be stopped. The dosage based on degree of vitreous seeding activity, determined clinically. The dosage intravitreal melphalan ranged from 20 to 33 mg based on clinical features from 2014 to 2016. However, once there was obvious ocular toxicity, a dose of 20 mg is routinely given, and doses over 30 mg were rarely used now. Therapeutic success was defined as the complete regression of all of the vitreous seeds without recurrence, while failure was defined as the persistence or recurrence of viable vitreous seeds.

### Chart review

The demographic details of the patients were collected, including the age at the time of the intravitreal melphalan injection. The data regarding the tumor features was collected at the time of the initial presentation, including the retinoblastoma stage (International Classification of Retinoblastoma) [17] and initial treatment method. The seed data included the seed classification at presentation (class 1 = dust, class 2 = sphere ± dust, or class 3 = cloud ± sphere or dust), and the final seed regression pattern (type 0 = not visible, type 1 = calcific, type 2 = amorphous, and type 3 = both types 1 and 2) [12]. The retinal toxicity was divided into five grades according to Munier’s report [13]. Furthermore, an abnormal hyaloid described by Azizet [18] was evaluated by B-scan ultrasonography (AVISO,France). After the completion of the treatment, the follow-up was extended based on the globe response. The outcome measures included vitreous seed control, treatment complications, and medication toxicity.

### Statistic al analysis

For the data analysis, the Statistical Package for the Social Sciences (SPSS) version 17.0 (SPSS Inc., Chicago, IL, USA) was used. The descriptive statistics included the means and standard deviations for all of the variables. The categorical variables were compared with the χ^2^ or Fisher’s exact test, while the continuous variables between the groups were compared with the Mann-Whitney U and Kruskal-Wallis tests. The Pearson’s or Spearman’s rank correlation was used to determine the relationships between the retinal toxicity grades and the other variables. A *p* value of < 0.05 was considered to be statistically significant.

## Results

In 23 patients, retinoblastoma was bilateral in 13(56.5%) patients and unilateral 10(43.5%) cases. 2 eyes was enucleated after intravenous chemotherapy instantly, 4 eyes was saved with cryotherapy treatments and transpupillary thermotherapy after intravenous chemotherapy, which were not included in the study. No case has familial history. The mean patient age at the time of the vitreous injection was 28 months (media*n* = 22 months, range = 12–50 months). At the time of the initial presentation, the affected eye was classified (International Classification of Retinoblastoma) described by Shields ^17^as group C (n = 2, 6.7%), group D (*n* = 19, 63.3%), or group E (*n* = 9, 30%). All of the eyes in this series had received previous intravenous chemotherapy (IVC) and/or intra-arterial chemotherapy (IAC). In addition, all of the eyes had viable vitreous seeding. The primary treatment included intravenous vincristine, etoposide, and carboplatin for 4–9 cycles, with an average of 6.3 (20/30), or IAC using a combination of melphalan and carboplatin for 2–6 cycles, with an average of 3.4 (13/30). In addition, 17 of the 30 eyes received 1–4 cryotherapy treatments, with an average of 2.6, while 25 of the 30 eyes received 1–5 transpupillary thermotherapy (TTT) therapy treatments, with an average of 2.7. The follow-up ranged from 9 to 36 months, with average of 21.2 ± 4.8 months,median was 24 months. The follow-up time was from the end of the injection course.

In total, 80 injections were administered in 30 eyes, with a mean of 2.7 sessions (median = 3, range = 1–5), at an interval of 2–4 weeks. Each eye received an intravitreal melphalan injection of 20–33 μg, with an average of 24.9 μg each time. For persistent seeds, the time of intravitreal injections is after 4.5 ± 0.95 cycles for systemic chemotherapy and 2.5 ± 0.92 cycles for intra-arterial chemotherapy. In recurrent cases, the time for time of intravitreal injections was immediately after recurrence or after intra-arterial chemotherapy.

Overall, the vitreous seeds were successfully controlled in 26 out of 30 eyes (86.7%).

Based on the morphological features, the vitreous seeds were classified as dust (*n* = 15), spheres (*n* = 10), and clouds (*n* = 5). The final seed regression patterns were classified as type 0 (*n* = 17), type 1 (*n* = 7), type 2 (*n* = 3), and type 3 (n = 3). Eleven of the eyes were spared from retinal toxicity. In the remaining eyes (*n* = 19), the retinal toxicity grades were classified from 1 to 5 (Table 1). There was no significant difference in the retinal toxicity (to the intravitreal melphalan) grades for the cloud, sphere, or dust seeding(*p* = 0.6798), in the seed regression patterns(*p* = 0.1852) or in abnormal hyaloidal interface(0.3672). There were no significant differences in the baseline or treatment characteristics between the groups of different grade retinal toxicity in mean number of IVC, mean number of the IAC treatments, mean number or cumulative dosage of the IVM injections (*p* > 0.05). However, mean dosage given showed significant difference between the groups.(*P* = 0.0045).

**Table 1:** Treatment characteristics between groups

	Clinical grading of retinal toxicity						ALL eyes	P value
	None	grade 1	grade 2	grade 3	grade 4	grade 5		
Number of eyes	11	4	3	3	3	6	30	
Mean number of IVC rounds(*n* = 20)	5.8	6.0	6.0	6.5	7.0	7.3	6.3	0.3013
Mean number of IAC rounds(*n* = 13)	3.3	3.5	3.0	3.5	4.0	3.0	3.4	0.9937
Mean number of IVM injections	2.7	3.0	2.3	3.0	2.3	2.8	2.7	0.8613
Mean dosage given (μg)	22.2	25.5	23.7	25.0	29.3	27.8	24.9	0.0045
Mean cumulative total dose(μg)	62.2	77.0	55.3	71.7	69.3	78.7	68.4	0.5306
Abnormal hyaloidal interface	1/11	0/4	1/3	1/3	1/3	1/6	5/30	0.3672

*IVC was conducted in 17 patients, while IAC was conducted in 10 patients. 3 patients recieved both IVC and IAC. The mean number was calculated in those patients who received the therapies. IVC intravenous chemotherapy, IAC intra-arterial chemotherapy, IVM intravitreal melphalan

**Fig. 1:**
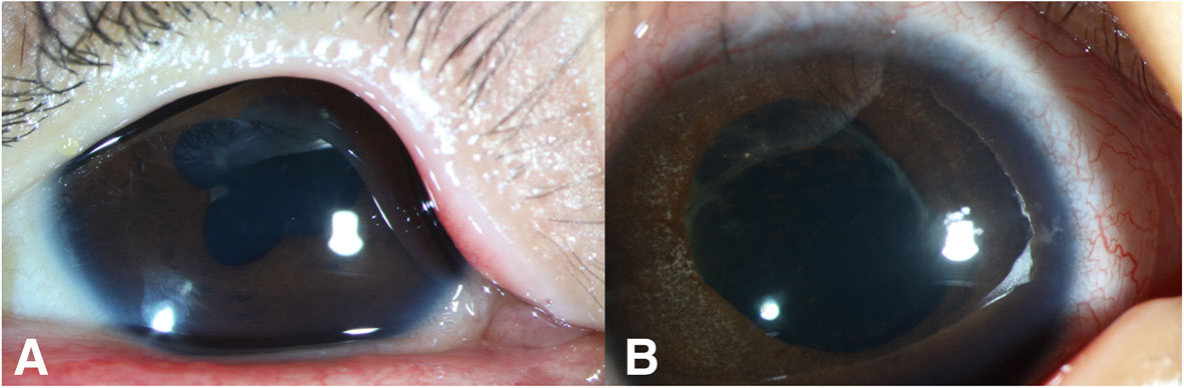
Iris and lens toxicity. One patient showed evidence of pupillary synechiae and a cataract (**a**). Another patient showed evidence of iris atrophy and received cataract surgery (**b**)

**Fig. 2:**
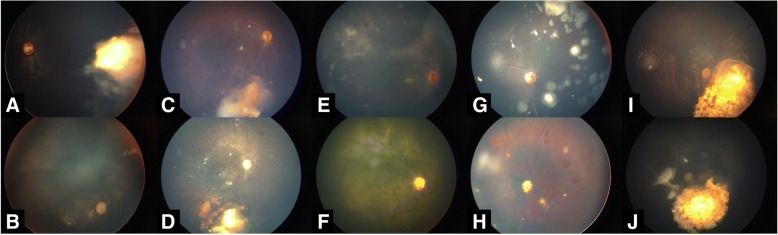
Panretinopathy and with optic atrophy. 5 cases with optic atrophy before and after IVM have been shown in Fig. 5.The recurrence of a retinoblastoma with diffuse vitreous seeds. (**a**) Eight months after three rounds of IAC and four injections showing panretinopathy with optic atrophy (**b**).Persistent seeds after three rounds of IAC.(**c**) Twelve months after three injections showing panretinopathy and vascular occlusion with optic atrophy.(**d**) Persistent seeds after five cycles of chemotherapy. (**e**)Four months after three doses of IVM injections showing complete control of vitreous seeds and vascular occlusion with optic atrophy.(**f**) Refractory diffuse vitreous seeds after six cycles of chemotherapy. (**g**) Three months after two doses of IVM injections showing complete control of vitreous seeds and hemorrhagic retinopathy with optic atrophy(**h**). Persistent seeds after three rounds of IAC(**i**) Eighteen months after two injections showing vascular occlusion with optic atrophy.(**j**)

**Fig. 3:**
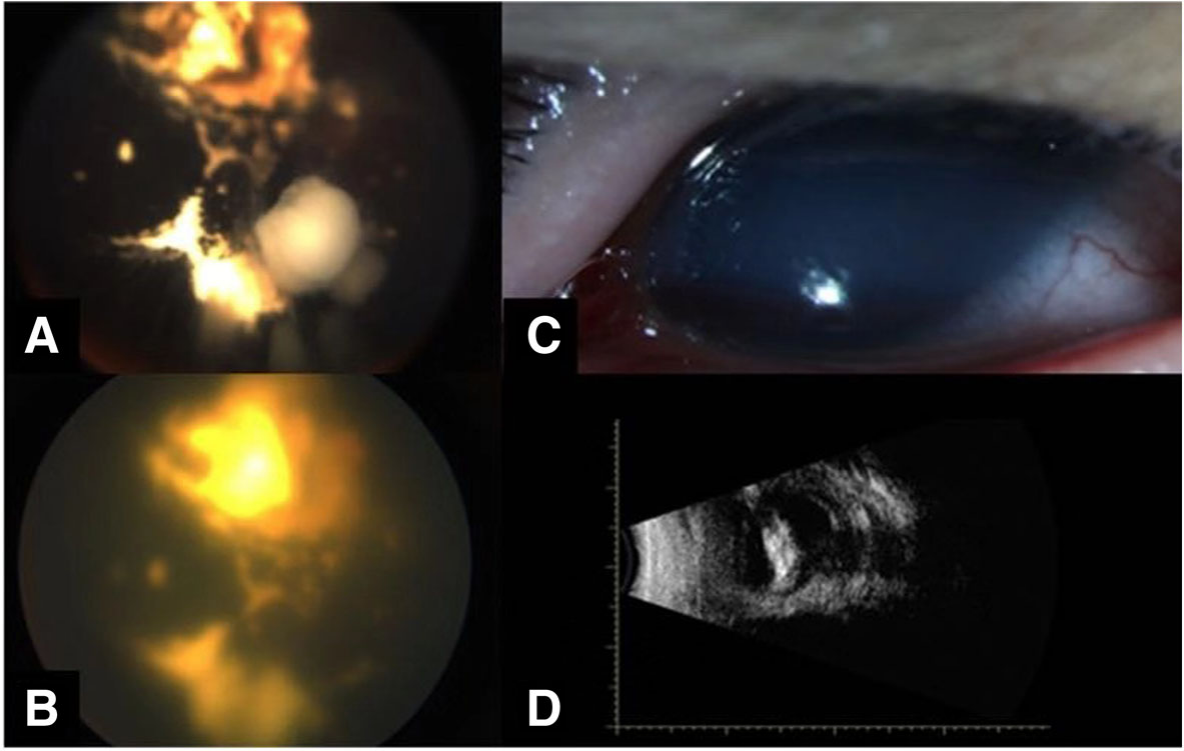
Corneal edema and phthisis. Refractory diffuse vitreous seeds in unilateral retinoblastoma after six cycles of intra-arterial chemotherapy. (**a**) After three doses of IVM showing partial regression. (**b**) Phthisis attributed to IVM with corneal edema and persistent hypotonia. (**c**) B-scan showed choroidal detachment and axial length was 16.5 cm.(**d**)

**Fig. 4:**
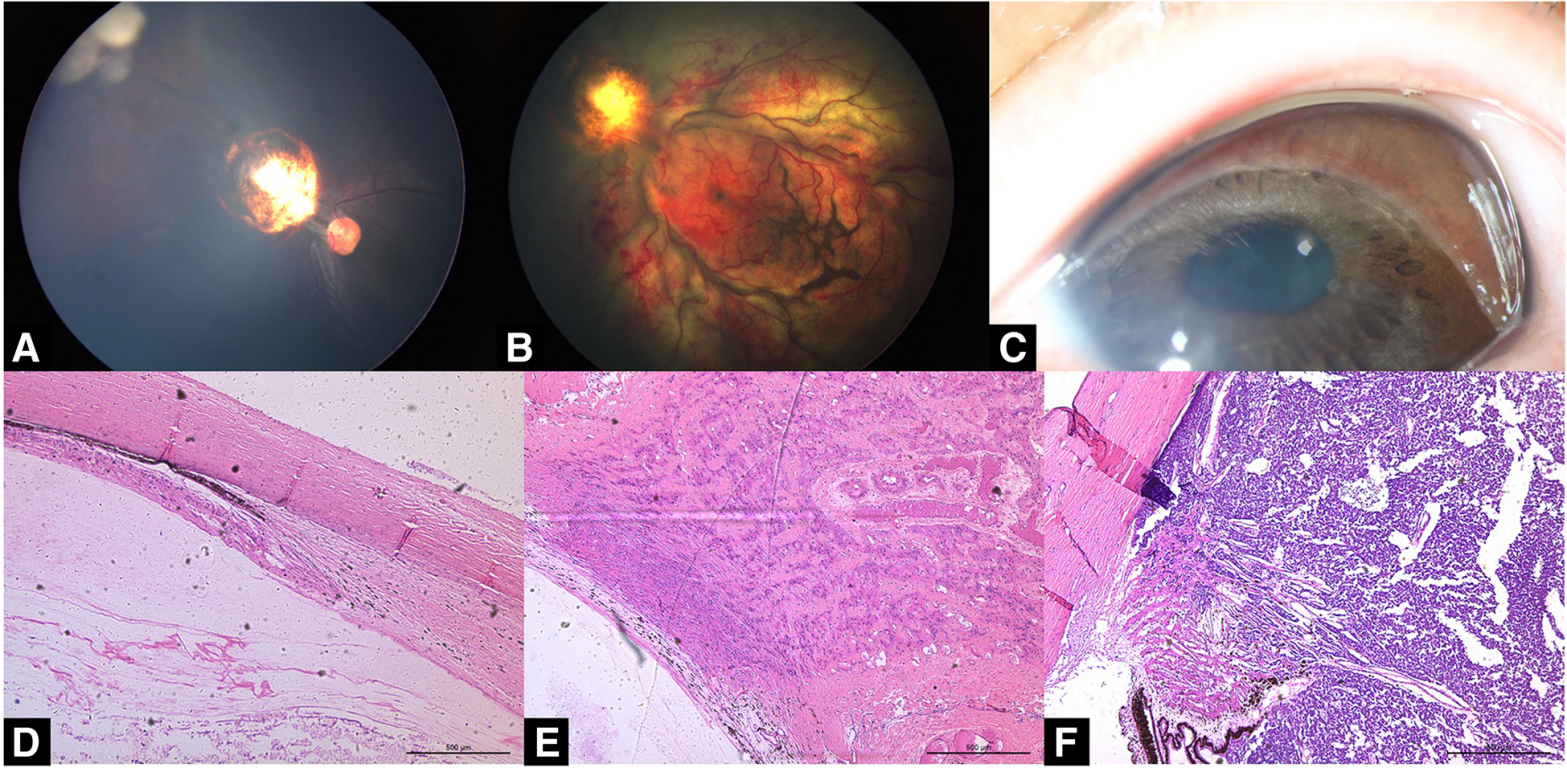
Retinal vessel toxicity. The recurrence of a retinoblastoma with a sphere of vitreous seeds was treated with two injections of intravitreal melphalan (30 μg) (**a**). The patient tolerated the initial injection well, with no apparent intraoperative or immediate postoperative complications. At the 1-week follow-up, the visual acuity was decreased to light perception, and the patient showed evidence of diffuse vascular occlusion with retinal edema and hemorrhage (**b**). At the 6-month follow-up, the patient showed evidence of pupillary synechiae, iris atrophy, and iris neovascularization (**c**). The fundus was not visible. At the 8-month follow-up, B-scan indicated the suspicious recurrence of the tumor. The eye was enucleated. The pathological findings revealed the severe atrophy of retina, choroid (**d**) and optic nerve (**e**) as well as extensive reactive gliosis with tumor invasion of ciliary body and choroid (**f**). (H&E, × 50)

**Fig. 5:**
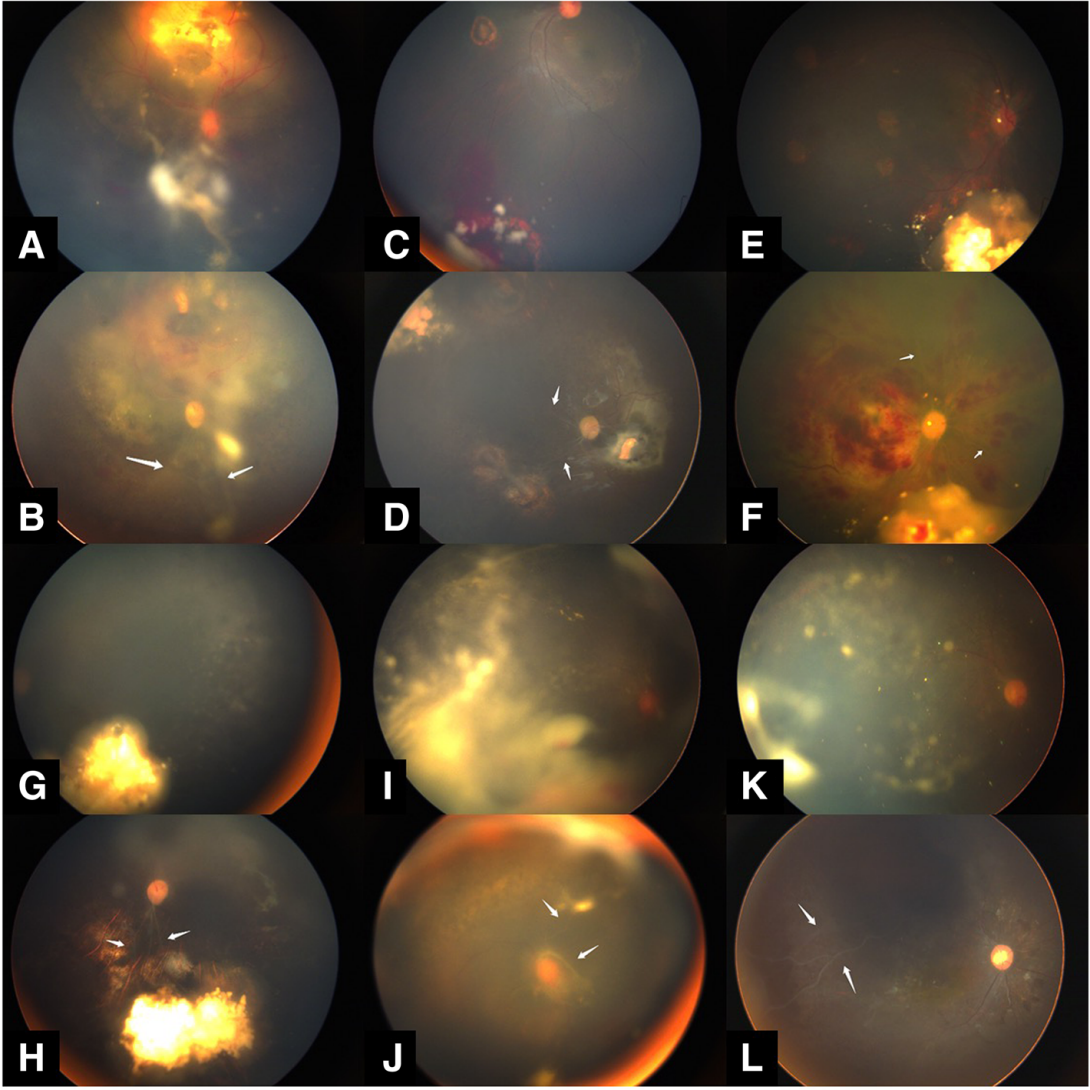
Vascular occlusion. 6 cases with vascular occlusion before and after IVM have been shown in Fig. 4. Persistent seeds after three rounds of IAC(**a**)Three months after three injections showing branch vascular occlusion(**b**). Refractory focal vitreous seeds after six cycles of chemotherapy(**c**). Six months after one injection showing branch vascular occlusion and retinopathy. (**d**) Persistent focal seeds after six cycles of intravenous chemotherapy and two IVM injections.(**e**) One month after another IVM injection showing hemorrhagic retinopathy and vascular occlusion.(**f**) Persistent seeds after six cycles of intravenous chemotherapy(**g**). Ten months after three injection showing branch vascular occlusion.(**h**) Refractory diffuse vitreous seeds after three rounds of IAC.(**i**) Eight months after five injection showing branch vascular occlusion and pupillary synechiae.(**j**)Persistent seeds after four rounds of IAC.(**k**) Eight months after four injection showing branch vascular occlusion and pupillary synechiae. (**l**) The vascular occlusions were marked by arrows

The complications included pupillary synechiae (13/30, 43.3%), iris atrophy (12/30, 40%), (Fig. 1) optic atrophy (6/30, 20%),(Fig. 2) vitreous hemorrhage (3/30, 10%), persistent hypotonia and phthisis bulbi, (4/30, 13.3%),(Fig. 3) retinal pigment epithelium (RPE) and choroidal atrophy (19/30, 63.3%), retinal vascular occlusion (12/30, 40.0%).(Fig. 4, Fig. 5) Twelve eyes demonstrated grade 3 or greater IVM-.

associated retinal or anterior segment toxicity post injection. Cataracts (8/30, 26.7%) were observed. Two of the eyes underwent cataract surgery 10 months and 12 months from the end of the injection course respectively. Five eyes resulted in enucleation: two eyes were removed due to persistent hypotony and phthisis bulbi (one eye still had active vitreous seeds), and three eyes was removed due to a retinal tumor and seed recurrence. The overall enucleation-free survival rate was 83.3% (25/30): 100% for group C, 89.5% for group D, and 66.7% for group E. There were no cases of extraocular extension or metastasis within the follow-up period.

## Discussion

Intravenous chemotherapy (chemoreduction) and IAC are currently the two most commonly used globe-conserving therapies for retinoblastoma cases. Vitreous seeds pose a notorious problem in retinoblastoma management, and the control of vitreous seeding can be challenging with both IVC and IAC. More recently, intravitreal melphalan has been found to be remarkably effective for the control of vitreous seeds. For example, Munier et al. [3, 16] reported the regression of vitreous seeds in 87% of the eyes treated with intravitreal melphalan. Shields et al. [11] reported therapeutic success with vitreous seed regression in all 11 eyes in their study with an intermediate dose of 20 to 30 μg.

Previous studies have focused mainly on Caucasian populations, but seldom on Asian populations, such as those in China and India, which have the largest numbers of retinoblastoma patients. In our study, we also reported many more complications than previously reported by other groups. Previously, Smith et al. [6] reported that the risk of intraocular toxicity appeared to be minimized through the use of melphalan at doses of less than or equal to 30 μg. However, we proposed that since the incidence of uveitis is higher in pigmented populations, especially in Asian countries [19], the uveitis inflammation and anterior ocular toxicity after an intravitreal melphalan injection would be more serious. Francis suggested increased toxicity in more deeply pigmented eyes [20],which was consistent with our findings as all of our patients are Chinese population that had more deeply pigmented eyes. In recent published article with Indian population, intravitreal melphalan showed complications including anterior chamber flare and cells, vitritis, synechiae formation and cataract, so Rao conducted intravitreal topotecan injection in the management of vitreous seeds [21]. As for the reason, Francis speculated that more deeply pigmented eyes may absorb increased levels of melphalan, resulting in more retinal pigment epithelium toxicity and, by extension, retinal and choroidal toxicity. A direct toxic effect of melphalan on RPE cells in vitro was found via morphological monitoring and toxicity assays, and may explain the clinical and angiographic RPE alterations observed in some retinoblastoma patients [22, 23]. Further studies that directly evaluate these mechanisms should be conducted, as well as studies into how inflammation might affect the iris, choroid, RPE, and retina. Chao et al. reported a case in which diffuse chorioretinal atrophy developed at the injection site after a single, low, standard 8 μg intravitreal melphalan injection [24]. In addition, Aziz et al. reported the occurrence of acute hemorrhagic retinopathy following an intravitreal melphalan injection for retinoblastoma [18]. From Asian population, Japanese researchers had reported chorio-retinal atrophy less than 1.5% after intravitreal injections, however standard dose of melphalan was 8 to 16 μg, which was much less than our study [25].With higher dose, better eye-retention rate was achieved in our research.

The number of injections and cumulative dose are not the risk factors, but single dose may be a risk factor, as mean dosage given showed significant difference between the groups. Reducing single dose of IVM injections probably help to reduce the toxicity. Optic atrophy and phthisis and are severe complications after IVM, while it is general believed that severe complications probably occur from doses of 50 mg [5]. Of course, the risk is likely to be overestimated. Many of the eyes received multiple treatments, such as intravenous chemotherapy, IAC, cryotherapy, and TTT, which may have aggravated the retinal toxicity. Cryotherapy may affect the function of ciliary body, resulting in persistent hypotonia. Furthermore, many cases with severe complications in this series received previous intra-arterial chemotherapy; we suggested that it may due to the cumulative vitreous dose of melphalan, which may have aggravated the retinal toxicity. Similarly, vascular occlusion were in part due to the dramatic regression of the tumor, because of the vascular toxicity from the delivery of higher concentrations of chemotherapy, when combined with repeated local treatments and increased cumulative dose of melphalan. Aziz proposed that the development of retinal toxicity most likely results from a retrohyaloid overdose [18]. However, it is not likely to be the etiology for all melphalan-related toxicity. In our cases, there was no significant difference in the retinal toxicity in abnormal hyaloidal interface.

Anterior segment abnormalities have been described extensively following intravitreal injections of anti-vascular endothelial growth factor agents [26, 27]. There is reason to propose this is due to anterior segment toxicity following the intravitreal melphalan injections. Francis [28] reported 5 cases (out of 76 patients) with anterior segment toxicity following intravitreal injections, which is rarely seen. We found that pupillary synechiae, iris atrophy, and cataracts occurred in 43.3, 40.0, and 26.7% of the patients, which were much more common than previously reported. The severity of inflammation could be related to the darkly pigmented iris of Asian Chinese. Instead, intravitreal topotecan appears effective and safe in controlling vitreous seeds in retinoblastoma [21].

This study was not without certain limitations. For instance, the number of participants included in this research was limited. Overall, the number of participants in each of the different groups might have been too few to reach statistical significance. There is no “control group” in this study, for all the patients were Chinese. We propose that multicenter prospective clinical study will carry more conviction in the future. Melphalan is a difficult drug to handle, requiring careful reconstitution and filtering, and administration within one hour of reconstitution. These precautions were met strictly in our study. We did find higher hates of both anterior segment and posterior segment complications than previously reported by other groups.

## Conclusion

In summary, intravitreal melphalan has allowed ophthalmic oncologists to salvage eyes that would have been enucleated, however, this does not come without both anterior and posterior segment toxicity. This is especially true for East Asian populations, which may suggest a connection with race. Unexpected retinal toxicity can occur even when the standard dose and a careful technique are employed, particularly in those eyes receiving multiple treatments.

## Abbreviations

IAC: Intra-arterial chemotherapy
IVC: Intravenous chemotherapy
IVM: Intravitreal melphalan
RPE: Retinal pigment epithelium
SPSS: Statistical Package for the Social Sciences
TTT: Transpupillary thermotherapy
UBM: Ultrasound biomicroscopy
